# Conservative Management of Mirizzi Syndrome in Community Hospital Setting

**DOI:** 10.7759/cureus.19144

**Published:** 2021-10-30

**Authors:** Lahari Vudayagiri, Omar F Mujahed, Logan Mellert, Rick Gemma

**Affiliations:** 1 General Surgery, Western Reserve Hospital, Cuyahoga Falls, USA

**Keywords:** mirizzi syndrome, cholecystocholedochal fistula, chronic cholelithiasis, t-tube

## Abstract

Mirizzi syndrome (MS) is a rare complication of chronic cholecystitis caused by the gallbladder wall compression of the common hepatic duct (MS1, based on McSherry classification) or as a cholecystocholedochal fistula (MS2). The incidence of MS in symptomatic cholelithiasis is very low. Patients often present with obstructive jaundice and right upper quadrant abdominal pain; symptoms not clinically unique from biliary colic or cholecystitis, and often misdiagnosed preoperatively.

We present the case of a 76-year-old female, initially diagnosed with chronic cholecystitis, who was found to have MS2 intraoperatively. She denied a prior history of abdominal surgery or biliary instrumentation. The patient underwent a subtotal cholecystectomy with common bile duct exploration, t-tube placement, and wide local drainage. She progressed well and was discharged home from the hospital on day seven with outpatient hepatobiliary surgery follow-up. At one-month follow-up, the patient had t-tube output of 200-300cc per day with remaining drains removed after having diminished output and no signs of biloma on CT. At the two-month follow-up, the patient had a minimal t-tube output with t-tube cholangiography showing contrast dye into the duodenum. Her t-tube was clamped and was removed at the three-month follow-up.

Surgical management of MS1 is generally laparoscopic or open cholecystectomy. Management of MS2 is complex and dependent on anatomic and pathologic factors. Surgical repair generally focuses on biliary-enteric reconstruction, with cholecystcholedochoduodenostomy or Roux-en-Y hepaticojejunostomy. Conservative surgical approach with subtotal cholecystectomy, common bile duct exploration, and biliary drainage is also reported as a safe alternative option. MS is a rare complication of chronic cholecystitis, and can be a cause of cholecystocholedochal fistula, which is often discovered intraoperatively during cholecystectomy; general surgeons should be familiar with conservative management of MS.

## Introduction

Mirizzi syndrome (MS) is a rare complication of gallbladder disease and comes in two subtypes. MS1 consists of extrinsic compression of the common hepatic duct or common bile duct by a stone while MS2 is when stones erode through the gallbladder wall forming a cholecystocholedochal fistula [1]. When large stones become impacted at the cystic duct or infundibulum, with chronic inflammation, erosion of the common bile duct can lead to this fistula.

Diagnostic imaging used in the diagnosis of MS range from CT, magnetic resonance cholangiopancreatography (MRCP), and endoscopic retrograde cholangiopancreatography (ERCP). Of these, MRCP is of most significance as it is noninvasive and provides multiple perspectives [2]. Again, since the classic presentation is often right upper quadrant (RUQ) abdominal pain, and rarely with jaundice and fever, it is often treated initially as cholecystitis or cholangitis and advanced imaging is often not obtained. Laboratory findings generally include elevations in serum alkaline phosphatase and bilirubin. 

If the diagnosis is made preoperatively, ERCP and sphincterotomy temporarily allow for biliary decompression and stent placement. However, optimal management is with cholecystcholedochoduodenostomy, or more commonly, Roux-en-Y hepaticojejunostomy by a hepatobiliary surgeon. However, conservative management of MS2 is essential for a general surgeon to know due to the high rate of misdiagnosis. As MS is commonly identified intraoperatively, it is essential for a general surgeon to know how to management MS conservatively to avoid catastrophic damage to the common bile duct (CBD) [1].

## Case presentation

A 76-year-old female presented for an elective cholecystectomy after being evaluated for cholelithiasis with pericholecystic fluid that was identified in ultrasound. She was diagnosed with chronic cholecystitis. The patient complained of some food intolerances and minimal tenderness of the RUQ abdomen. The patient has a history of hypertension, type 2 diabetes, osteopenia, obesity, diverticulosis, hyperparathyroidism, osteoarthritis (OA) of knees, and hyperlipidemia. The patient's family history consists of hypertension (HTN), diabetes mellitus (DM), cerebral vascular accident (CVA), in the patient’s mother. Surgical history is comprised of parathyroidectomy, pericardial window, colonoscopy with polypectomy, and left total knee arthroplasty. 

**Figure 1:**
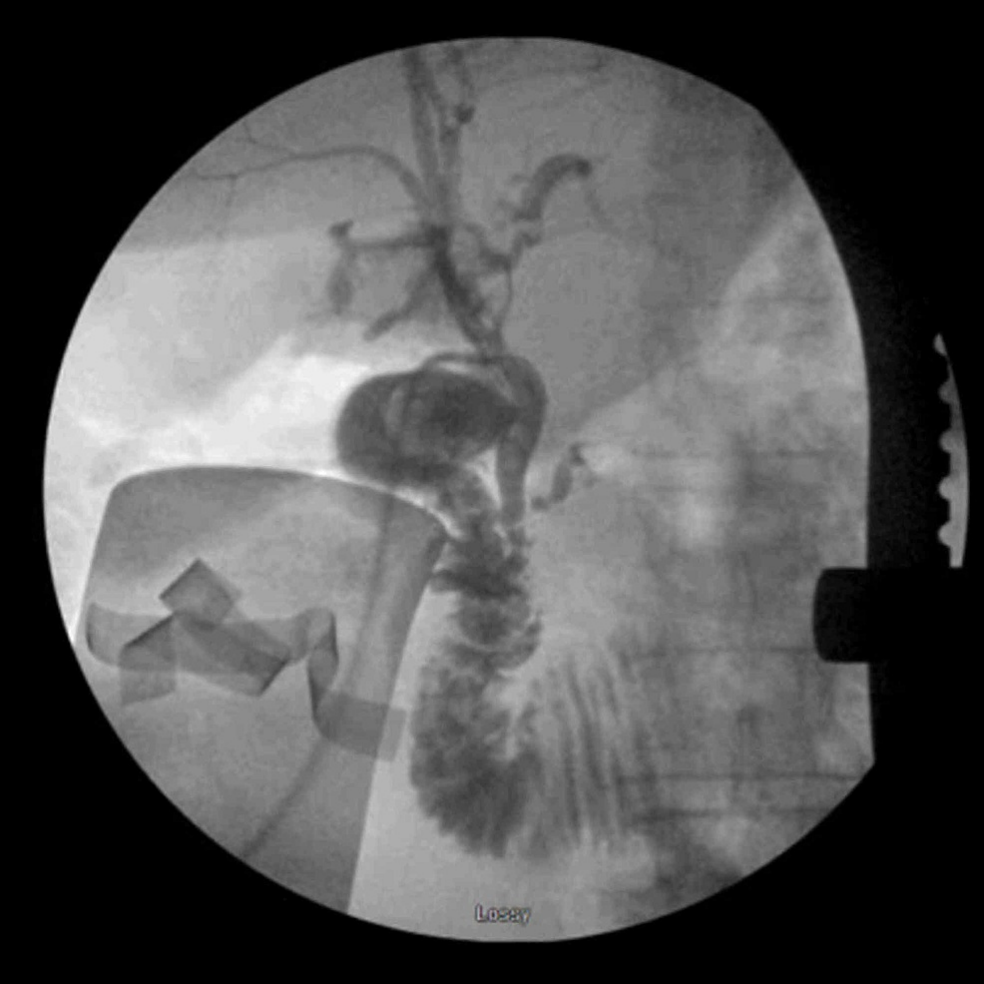
Intraoperative t-tube cholangiogram showing proximal CBD filling defect as well as extravasation of dye and filling into the gallbladder. CBD: common bile duct

A laparoscopic cholecystectomy was initially the plan of choice. During dissection of the gallbladder, significant scarring was noted and a structure entering the gallbladder anterior to the cystic duct was observed, which was carefully dissected and a ductotomy was performed. Intraoperative cholangiogram through the duct showed proximal CBD filling defect with extravasation of dye. Due to a possible CBD ductotomy, the procedure was converted to open. The ductotomy was within the CBD which was further explored and flushed and did not show any evidence of stones within the duct but did show amounts of biliary sludge and sediment. A t-tube was inserted into the ductotomy and intraoperative t-tube cholangiogram revealed the ductotomy within the CBD and dye extravasating into the gallbladder itself (Figure 1). After the cystic duct was visualized and clipped, the gallbladder was dissected from the liver bed and opened to further visualize the CBD communication with the gallbladder. The t-tube was seen passing through the infundibulum of the gallbladder with clear visualization of a long fistula tract between the CBD and gallbladder itself. The remainder of the gallbladder was closed over a Blake drain and a Jackson-Pratt (JP) drain was inserted in the infrahepatic space. 

**Figure 2:**
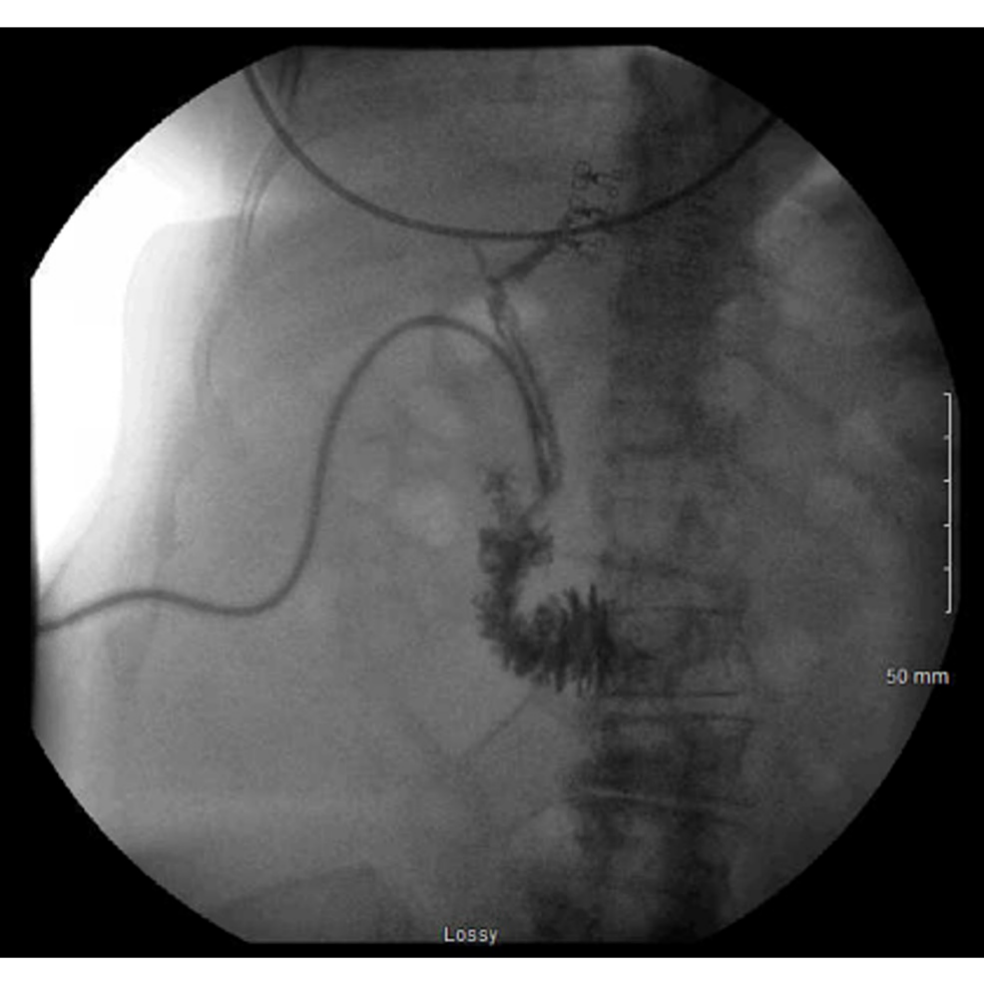
Two months postoperative t-tube cholangiogram showing satisfactory draining of CBD with no filling defects and patent biliary tree. CBD: common bile duct

Postoperative management consisted of strict ins and outs of drains and assessing the patient’s clinical examinations and labs. The patient was discharged postoperative delirium (POD) 7 when she was tolerating a regular diet, with decreased bilious output, and liver function tests were normalized. At one-month outpatient follow-up, the patient had 200-300cc per day drainage from the t-tube and about 50cc drainage each from the other two drains. At this time, the patient had a CT abdomen taken and showed no evidence of biloma or biliary dilatation. Both the JP and Blake drains were removed at this time. At the two-month postoperative, the t-tube had less than 5 cc output in the t-tube study performed at this time showed contrast into the duodenum (Figure 2). The t-tube was then clamped and removed three months postoperatively. The patient’s drain and incision sites were well healed and closed at the three-month postoperative visit. 

## Discussion

**Figure 3:**
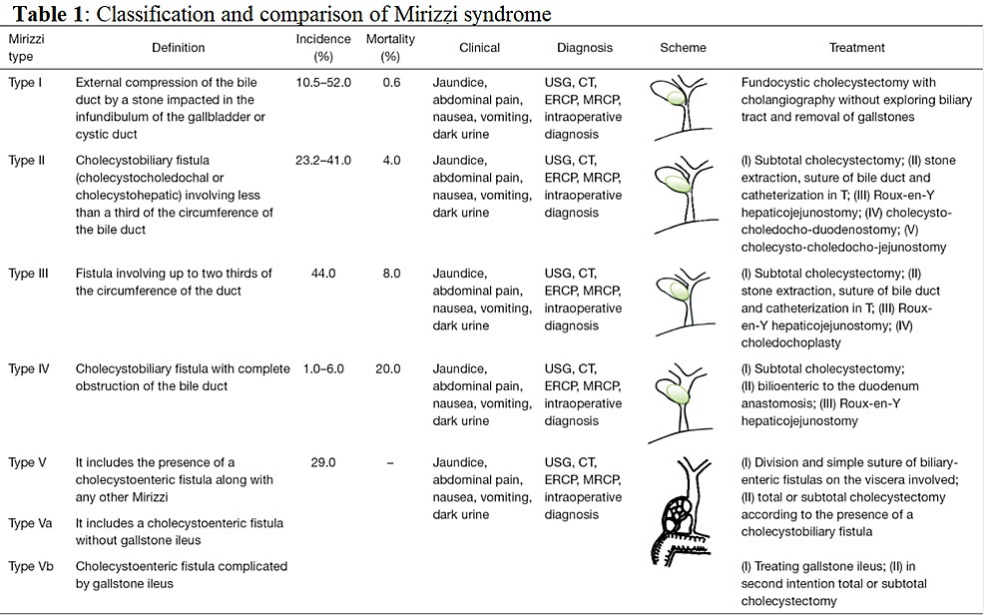
Csendes classification and comparison of Mirizzi syndrome (data collected from 1997 to 2015). The image is reprinted from Valderrama-Treviño et al. [4]. MRCP: magnetic resonance cholangiopancreatography; ERCP: endoscopic retrograde cholangiopancreatography

Mirrizzi syndrome is commonly classified through two different classification systems: McSherry et al. and Csendes et al. McSherry classification consists of MS1 which is external compression of the common hepatic duct, which is equivalent to Csendes classification MS1. MS2 is described as gallbladder stone compression against the gallbladder wall causing fistulation of the gallbladder and the hepatic and/or CBD. McSherry MS2 classification encompasses Csendes et al. classification type 2-4. Type 5 was added to Csendes classification in 2007 and encompasses the presence of a cholecystoenteric fistula along with any other MS with or without gallstone ileus (Figure 3) [3]. MS occurs secondary to chronic inflammation of the gallbladder causing inflammatory, edematous tissue which becomes fibrotic to nearby structures causing erosion of the mucosa in between and fistula formation. Although carcinoma of the gallbladder is rare, a frozen section of the gallbladder wall should be sent in all cases. The pathology for our patient showed no evidence of carcinoma, however, presence of chronic cholecystitis. 

Unfortunately, there are no pathognomonic signs to MS and it often presents similar to cholecystitis. Although MRCP and ERCP are considered the most sensitive and specific tests if suspecting MS, more than 50% of the diagnoses are made intraoperatively [5]. Hence, as presented in this case, it is essential for all general surgeons to be knowledgeable about conservative management of MS to avoid major injuries of the CBD [1]. As presented in this case report, a subtotal cholecystectomy with t-tube placement is appropriate management of MS2 without the need for hepatobiliary intervention. Other treatment options suggested are choledochoduodenostomy, and right hepaticojejunal Roux-en-Y [1,2,4,6]. A choledochoduodenostomy study shows that there are 20.5% early-stage complications compared to the t-tube placement that was 18.4% [7]. If the biliary confluence is intact as well as vascular structures, the best results are seen with a hepaticojejunal Roux-en-Y [8]. However, as demonstrated in this case report, conservative management of MS2 should be a familiar procedure to all general surgeons.

## Conclusions

With the incidence being very low, MS2 can be misdiagnosed but with preoperative preparedness, general surgeons can manage MS2 without referral to the hepatobiliary. Imaging modalities include US, ERCP, and MRCP, where MRCP is the best to appreciate a change in the normal anatomy and ERCP has the highest sensitivity. Subtotal cholecystectomy with t-tube placement is an acceptable way of conservative treatment measure for MS2. Other measures to treat MS2 include choledochoduodenostomy or Roux-en-Y hepaticojejunostomy, where the latter is preferred if biliary confluence is intact as well as vascular structures.
